# Three-way group decisions using evidence theory under hesitant fuzzy linguistic environment

**DOI:** 10.1038/s41598-023-49086-1

**Published:** 2023-12-20

**Authors:** Wenke Ding, Xingchen Li, Xiajiong Shen

**Affiliations:** 1 School of Computer and Information Engineering, Henan University, Kaifeng, 475004 China; 2 Henan Engineering Research Center of Intelligent Technology and Application, Henan University, Kaifeng, 475004 China

**Keywords:** Information technology, Computer science

## Abstract

In the actual decision-making process, there will be situations where decision-makers with hesitant attitudes have difficulties in evaluating alternatives numerically, and hesitant fuzzy linguistic term sets can provide decision-makers with an effective way to describe hesitancy in linguistic terms. In multi-attribute group decision-making, each decision maker typically holds different preferences. If the variation in decision makers’ assessment weights across evaluations of each attribute for every alternative is not adequately accounted for, it can result in a problem of coarse-grained calculations, leading to information loss. Additionally, the three-way decision model faces significant challenges in information fusion within the context of the hesitant fuzzy linguistic environment. Therefore, we propose a new three-way decision-making model under the hesitant fuzzy linguistic environment. The model obtains the confidence of different decision makers in attribute evaluations through the fusion of D-S evidence theory, and can perform more fine-grained fusion calculations on the evaluation information of different decision makers. In addition, the model considers the cost function of each alternative in different decision-making actions under hesitant fuzzy linguistic environment, calculates the two thresholds of each alternative in the three-way decision model, and derives the decision rules. The effectiveness of the model is verified through a numerical example and two comparative experiments, therefore, the model can be applied in intelligent classification or recommendation systems of hesitant fuzzy linguistic information systems.

## Introduction

In real life, decision-makers regularly generate uncertain information, thus the research of decision-making methods under the uncertain environment has received more and more attention^1–3^. Three-way decision (3WD) is a powerful methodology to tackle the decision-making issue of uncertain and imprecise data by means of rules generation, and the theory is still expanding rapidly at present. The 3WD theory was originally proposed by Yao^4^ initially to give a reasonable explanation of the three regions in Decision Theory Rough Sets (DTRSs). For the three regions, they are interpreted as acceptance, non-commitment, and rejection three actions, respectively. Since it was proposed, many scholars^5^ in different fields have expanded the theory and applied it in practice. For example, Yao et al.^6^ utilized the 3WD method for medical decision support and treatment plan decision. Zhang et al.^7^ presented a sequential 3WD model to address the decision-making issue with attribute increments. Lang et al.^8^ presented a three-way group conflict analysis model using Pythagorean fuzzy sets and the 3WD method for the treatment of conflict problems in multi-expert evaluations. Yao et al.^9^ provided a 3WD model on the basis of a ranking relation method to solve multi-attribute decision problems. There is a wide variety of fields in which the 3WD theory has been applied, including solid waste management^10^ and green supplier selection^11^, project resource allocation^12^, person-job fit^13^ and so on. In addition, given the wide application and good performance of artificial intelligence technology in various fields^14–20^, Li et al.^21^ combined the 3WD model with a deep neural network and applied it in the field of image data analysis.

Multi-attribute group decision making (MAGDM)^22^ is an important research component in the field of decision-making, which can exploit the advantages of analyzing multiple attributes in multi-attribute decision making (MADM)^23^ and the fusion of multiple decision makers’ assessments in group decision-making (GDM)^24^. MAGDM is able to fuse the preferences of multiple decision makers for alternatives and obtain a ranking relationship of a set of alternatives through some decision mechanism^25^. In practical decision-making issues, due to the differences in the description of uncertainty problems by decision-makers, the MAGDM model is usually combined into various uncertain environments^11^. For example, Liang et al.^26^ utilize the hesitant probability fuzzy sets to describe decision makers’ preferences in decision-making, and proposed a MAGDM method with hesitant probability fuzzy sets. Using interval-valued intuitionistic fuzzy sets, Liu et al.^27^ developed a hybrid MAGDM method. Yu et al.^28^ considered the differences in culture and knowledge background of decision-makers in an uncertain environment, and proposed a consistency model in the MAGDM problem to help decision-makers reach consensus. In addition, the use of linguistic terms by decision-makers on certain issues in real life can better express the hesitancy and uncertainty in the evaluation of things, such as the issue of movie evaluation, etc. Rodríguez^29^ proposed a hesitant fuzzy linguistic term set (HFLTS), it enables evaluator to represent the hesitancy in assessments through hesitant fuzzy linguistic terms^11,30^. In subsequent developments, researchers considered the relationship between decision makers and linguistic term sets from different perspectives. Zhang et al.^31^ considered the situation where decision makers use different linguistic term sets for evaluation, and proposed a two-sided matching decision making method, thus giving decision makers a more flexible evaluation method. Li et al.^32^ established a personalized individual semantics learning model to obtain personalized numerical scales of linguistic terms for decision makers.

How to effectively aggregate the evaluation information of multiple decision-makers is also a subject of great concern in MAGDM. In the hesitant fuzzy linguistic information system, most of the current aggregation schemes fuse the evaluation of multiple decision makers through aggregation operators. However, this method will cause information loss during the aggregation process^33,34^, for instance, decision makers’ personal preferences will be lost in the calculation process^33^. The occurrence of such losses is primarily due to the fact that the weights assigned to decision makers are typically taken into account in the evaluation of each attribute of every alternative. However, each decision maker’s assessments may have varying weights compared to other decision makers. If all of a decision maker’s assessments are calculated using a fixed weight, it can lead to a loss of precision in their evaluations, resulting in an issue of information loss. To address this problem, researchers have introduced Dempster-Shafer evidence theory (DSET)^35^ to integrate the uncertain decision information between experts. Compared with the aggregation operator method, DSET can reduce information loss in multi-attribute group information fusion. This is based on the concept of DSET, where each decision maker’s assessment of an attribute is treated as evidence, and a more granular calculation of decision maker assessments is performed based on the confidence in the evidence. Therefore, we combine DSET with the 3WD method to fuse the evaluation preferences of multiple decision-makers under the HFL environment. In addition, more and more researchers are currently exploring the problems of information loss and information fusion in fuzzy information systems based on behavioral decision-making theory^36–39^.

In the general 3WD method, the deduction of decision rules needs the support of conditional probability, so the evaluation of conditional probability is a crucial problem. At present, the conditional probabilities in many three-way decision models are obtained through the calculation of equivalence class and information table^40^. The computation of equivalence classes needs to satisfy certain equivalence relations and requires decision attributes as one of the prerequisites^11,41,42^. However, there may be no decision attribute in the actual decision information tables. Therefore, as a method that can evaluate the relative pros and cons of all the objects in the information table, the TOPSIS method has been applied in many 3WD models. For instance, Du et al.^43^ through TOPSIS and grey incidence analysis proposed a grey multi-criteria 3WD model. Zhang et al.^44^ use the TOPSIS method and 3WD theory to construct a classification and ranking decision method. At the same time, it also achieved good results in diverse fuzzy sets^45–49^. Thus, due to the effectiveness of the TOPSIS method, we try to use it to calculate conditional probabilities in evidence-based information systems.

However, the research on the 3WD model under the HFL environment has rarely addressed the decision maker’s personal preferences and the connections between decision-makers. To reduce the loss of information fusion among decision makers and improve the group decision-making ability of 3WD under an HFL environment, we introduce Dempster-Shafer evidence theory and TOPSIS method into the 3WD theory. Overall, we construct a new 3WD model that can solve the evaluation problem in the HFL context with the help of evidence theory and the TOPSIS method. First, we assess the conditional probability in the HFL information system through the TOPSIS method based on evidence theory. Then, we consider the HFLTS cost function for each alternative and calculate the corresponding thresholds. At last, we present the whole derivation process of the 3WD decision rules.

The main points of the remaining subsections of this paper are summarized as follows. Concepts related to the 3WD, HFLTSs, and DSET, are reviewed in “Preliminaries” section. “Three-way decision with HFLT expression of loss functions” section constructs the 3WD model with HFLT expression of cost functions. “The estimation of conditional probability” section utilizes the HFL TOPSIS method based on DSET to estimate the conditional probability. In “The decision-making steps and the algorithm“ section, an illustrative example of a green supplier’s selection problem is given to solve the hesitant fuzzy MAGDM problem by using our model. In Section 6, we analytically illustrate the effectiveness and applicability of the proposed model through a comparative experiment of two examples. “Illustrative example” section sums up the proposed method and discusses future research and expansion.

## Preliminaries

### Three-way decision

As a result of Yao’s insight^4^, Bayesian risk decision-making was introduced into rough sets, and the classic three-way decision model was proposed^40^. Similarly, in the classical 3WD model there is a set of states and a set of actions^40,50^ according to the degree of conformity of the alternatives to expectations and the decisive actions taken, respectively. Suppose the set of alternatives $U=\left\{ {{u}_{1}},{{u}_{2}},...,{{u}_{m}} \right\}$ is a set with *m* objects to be decided, and two states $\Omega =\left\{ X,\lnot X \right\}$ for each alternative $u_i\in U$ can be interpreted as $u_i$ being in X and not in X, respectively.The set of actions $\mathscr {A}=\left\{ {{a}_{P}},{{a}_{B}},{{a}_{N}} \right\}$ denotes the decision behavior in taking actions ${{a}_{P}}$, ${{a}_{B}}$ and ${{a}_{N}}$ on alternatives $u_i\in U$ classified to the positive region *POS*(*X*), the boundary region *BND*(*X*) and the negative region *NEG*(*X*), i.e., denoted as taking the decisions of accepting, deferring and rejecting the alternatives.The decision costs of taking action ${{a}_{P}}$, ${{a}_{B}}$ and ${{a}_{N}}$ when the alternatives belong to state *X* are ${\psi }_{PP}$, ${\psi }_{BP}$ and ${\psi }_{NP}$, respectively; the decision costs of taking the same actions when an alternative belongs to state $\lnot X$ are ${\psi }_{PN}$, ${\psi }_{BN}$ and ${\psi }_{NN}$, respectively. The expected cost $\mathbb {E}({a}_{ \vartriangle } |X)(\vartriangle =P,B,N)$ for each alternative $u_i\in U$ is calculated by the following formula:

1 $$\begin{aligned}&\mathbb {E}({a}_{P}|u_i)=\psi _{P P}Pr(X |u_i)+\psi _{P N}Pr(\lnot X | u_i), \nonumber \\&\mathbb {E}({a}_{B}|u_i)=\psi _{B P}Pr(X | u_i)+\psi _{B N}Pr(\lnot X | u_i),\nonumber \\&\mathbb {E}({a}_{N}|u_i)=\psi _{N P}Pr(X | u_i)+\psi _{N N}Pr(\lnot X | u_i). \end{aligned}$$

where $Pr(X|u_i)$ denotes the conditional probability of $u_i$ belongs to state *X*. Which satisfies the condition $Pr(X|u_i) + Pr(\lnot X|u_i) = 1$.

In view of the general process of Bayesian risk decision-making, the minimum cost decision rule can be derived as follows:

2 $$\begin{aligned}&\text{(P0) } \ \text{ If } \ \mathbb {E}({a}_{P}|u_i)\le \mathbb {E}({a}_{B}|u_i),\mathbb {E}({a}_{P}|u_i)\le \mathbb {E}({a}_{N}|u_i),\text{ dicide }\ u_i\in POS(X); \nonumber \\&\text{(B0) } \ \text{ If } \ \mathbb {E}({a}_{B}|u_i)\le \mathbb {E}({a}_{P}|u_i),\mathbb {E}({a}_{B}|u_i)\le \mathbb {E}({a}_{N}|u_i),\text{ dicide }\ u_i\in BND(X);\nonumber \\&\text{(N0) } \ \text{ If } \ \mathbb {E}({a}_{N}|u_i)\le \mathbb {E}({a}_{P}|u_i),\mathbb {E}({a}_{N}|u_i)\le \mathbb {E}({a}_{B}|u_i),\text{ dicide }\ u_i\in NEG(X). \end{aligned}$$

According to the actual semantic interpretation of the cost functions, we suppose that ${\psi }_{PP}\le {\psi }_{BP} < {\psi }_{NP}$ and ${\psi }_{NN}\le {\psi }_{BN} < {\psi }_{PN}$. In addition, considering the condition $Pr(X|u_i) + Pr(\lnot X|u_i) = 1$, the above decision rules can be simplified as:

3 $$\begin{aligned}&\text{(P1) } \ \text{ If } \ Pr(X|u_i) \ge \alpha ,\text{ dicide }\ u_i\in POS(X); \nonumber \\&\text{(B1) } \ \text{ If } \ \beta< Pr(X|u_i) < \alpha ,\text{ dicide }\ u_i\in BND(X); \nonumber \\&\text{(N1) } \ \text{ If } \ Pr(X|u_i) \le \beta ,\text{ dicide }\ u_i\in NEG(X). \end{aligned}$$

where

4 $$\begin{aligned} \alpha =\frac{\psi _{P N}-\psi _{B N}}{\left( \psi _{P N}-\psi _{B N}\right) +\left( \psi _{B P}-\psi _{P P}\right) },\nonumber \\\beta =\frac{\psi _{B N}-\psi _{N N}}{\left( \psi _{B N}-\psi _{N N}\right) +\left( \psi _{N P}-\psi _{B P}\right) }. \end{aligned}$$

### Hesitant fuzzy linguistic term sets

Suppose $S = \{ {s}_{\gamma } | \gamma =$-$h, \cdots , 0,\cdots , h \}$ is a linguistic term set (LTS)^51^, which is a discrete finite set with 2h + 1 elements, where each element $s_{\gamma }$ in the set denotes a linguistic term (LT). For instance, when h = 3, a LTS with seven linguistic terms can be obtained, $S = \{ s_{\text {-3}}: absolutely \ dislike,\ s_{\text {-2}}: dislike, \ s_{\text {-1}}: a \ little \ dislike,\ s_{0}: medium, \ s_{1}: a \ little \ like,\ s_{2}: like,\ s_{3}: absolutely \ like \}$. In addition, LTSs also has the following properties. Let $s_a$ and $s_b$ be two LTs. Then,

- Max operator: $$max(s_{a},s_{b}) = s_{a},\ if \ s_{a} > s_{b}$$.
- Min operator: $$min(s_{a},s_{b}) = s_{a},\ if\ s_{a} < s_{b}$$.
- The set is ordered: $s_{a}>s_{b} \Leftrightarrow a > b$.

Considering the hesitancy and uncertainty that experts have shown in the assessment process, HFLTSs were proposed as an effective way to express uncertainty to solve this issue by Rodriguez et al.^29^.

#### Definition 1

^29^ Let $S = \{ {s}_{\gamma } | \gamma =$-$h, \cdots , 0,\cdots , h \}$ be a LTS. A finite ordered subset of successive LTs in *S* constitutes an HFLTS $H_S$ on *S*.

Subsequently, the definition of HFLTSs was further extended with the concrete mathematical formulation by Liao et al.^52^.

#### Definition 2

^52^ Let *V* be a finite and non-empty universe, $S = \{ {s}_{\gamma } | \gamma =$-$h, \cdots , 0,\cdots , h \}$ be a LTS. Then an HFLTS can be defined as $H_S = \{<x, h_S (x)>| \ x \in V \}$, where

5 $$\begin{aligned} h_{S}(x)= \{s_{\gamma _{l}} \mid s_{\gamma _{l}} \in S,\ l=1,2, \cdots , L;\ \gamma _{l} \in \{\text {-}h,...,0,...,h \} \}. \end{aligned}$$

where $h_{S}(x)$ is an HFLE which can indicate the degree of conformity of the object $x \in V$ belonging to the set $H_S$, $s_{\gamma _{l}}$ are the continuous terms in *S*, *L* is the number of LTs in $h_{S}(x)$.

#### Definition 3

^53^Let $S = \{ {s}_{\gamma } | \gamma =$-$h, \cdots , 0,\cdots , h \}$ be a LTS. The linguistic terms can be converted into membership degree of equivalent information through the transformation function g :

6 $$\begin{aligned} g:[\text {-}h,h] \rightarrow [0,1],\ g( s_{\gamma _{l}})= \frac{(\gamma _{l} + h)}{2h}. \end{aligned}$$

#### Definition 4

^30^ Let *V* be a finite and non-empty universe, $S = \{ {s}_{\gamma } | \gamma =$-$h,\cdots ,0,\cdots ,h \}$ be a LTS, and $h_{S}(x)= \{s_{\gamma _{l}} \mid s_{\gamma _{l}} \in S, l=1,2, \cdots , L;\ \gamma _{l} \in \{\text {-}h,\cdots ,0,\cdots , h \} \}$ be a HFLE. The expectation value of $h_{S}(x)$ can be defined as:

7 $$\begin{aligned} G(h_{S}(x)) = \frac{1}{L}\sum _{l=1}^L g(s_{\gamma _{l}}). \end{aligned}$$

#### Definition 5

^30^ Let *A*, *B* be two HFLTSs and $x\in$ *V*, the following comparison rules are introduced for two HFLEs $h_{S_A}$ and $h_{S_B}$:

8 $$\begin{aligned}&(1) \ \text{ If } \ G(h_{S_A}(x))> G(h_{S_B}(x)),\ then \ h_{S_A}(x) > h_{S_B}(x); \nonumber \\&(2) \ \text{ If } \ G(h_{S_A}(x))< G(h_{S_B}(x)), \ then \ h_{S_A}(x) < h_{S_B}(x);\nonumber \\&(3) \ \text{ If } \ G(h_{S_A}(x)) = G(h_{S_B}(x)), \ then \ h_{S_A}(x) = h_{S_B}(x). \end{aligned}$$

#### Definition 6

Translation function $T_{G_H}$^29^. Let $S = \{ {s}_{\gamma } | \gamma = \ \text {-}h,...,0,...,h \}$ be a LTS, the translation rules from linguistic expressions to HFLE are as follows:

- $$T_{G_{H}}\left( s_{\gamma }\right) =\{s_{\gamma } \mid s_{\gamma } \in S \}$$;
- $T_{G_{H}}$ (less than $\left. s_{\gamma }\right) =\left\{ s_{\beta } \mid s_{\beta } \in S\right.$ and $\left. s_{\beta }<s_{\gamma }\right\}$;
- $T_{G_{H}}$ (at most $\left. s_{\gamma }\right) =\left\{ s_{\beta } \mid s_{\beta } \in S\right.$ and $\left. s_{\beta } \le s_{\gamma }\right\}$;
- $T_{G_{H}}$ (greater than $\left. s_{\gamma }\right) =\left\{ s_{\beta } \mid s_{\beta } \in S\right.$ and $\left. s_{\gamma }<s_{\beta }\right\}$;
- $T_{G_{H}}$ (at least $\left. s_{\gamma }\right) =\left\{ s_{\beta } \mid s_{\beta } \in S\right.$ and $\left. s_{\gamma } \le s_{\beta }\right\}$;
- $T_{G_{H}}$ (between $s_{\alpha }$ and $\left. s_{\beta }\right) =\left\{ s_{\gamma } \mid s_{\gamma } \in S\right.$ and $\left. s_{\alpha } \le s_{\gamma } \le s_{\beta }\right\}$

### Dempster-Shafer theory of evidence

As an uncertain reasoning approach, Dempster Shafer evidence theory (DSET)^35^ has certain advantages in fusing uncertain multi-source information^54^. In addition, DSET has been used in various domains, for instance, gesture recognition^55^, fault diagnosis^56^, and so on.

#### Definition 7

^57^ Let $\Theta = \{ {\theta }_{1},{\theta }_{2},...{\theta }_{n} \}$ be a frame of discernment (FOD) and $2^\Theta$ be the power set of $\Theta$. The basic probability assignment(BPA) function demotes a mapping relationship *m*: $2^\Theta \rightarrow [0,1]$ and satisfies the following conditions.

9 $$\begin{aligned} \sum _{\xi \in 2^\Theta } m(\xi )=1 \text{ and } m(\varnothing )=0. \end{aligned}$$

where $\xi$ is a subset of $2^\Theta$, $\varnothing$ is an empty set, and $m(\xi )$ indicates the belief in set $\xi$ of a proposition based on the current environment. For proposition $\xi$ of $m(\xi )>0$, it is regarded as a focal element in the evidence theory. The probability $m(\Theta )$ assigned to $\Theta$ can be denoted as the degree of ignorance. It means the probability remaining after assigning all subsets of $\Theta$. A body of evidence *M* consists of all focal elements, it is represented as follows:

10 $$\begin{aligned} M = \left\{ (\xi , m(\xi )) \mid \xi \in 2^{\Theta }, m(\xi )>0\right\} . \end{aligned}$$

#### Definition 8

^57^ Suppose $M_1$ and $M_2$ be two independent evidences derived by the same frame of discernment $\Theta$, the fuse between them is indicated by $M=M_1 \oplus M_2$, the evidence combination rules are as below:

11 $$\begin{aligned} m(\xi )= & {} \frac{1}{1-K} \sum _{\xi _{1} \cap \xi _{2}=\xi } m_{1}\left( \xi _{1}\right) m_{2}\left( \xi _{2}\right) ,\xi \ne \varnothing , \ m(\varnothing )=0. \end{aligned}$$

12 $$\begin{aligned} K= & {} \sum _{\xi _{1} \cap \xi _{2}=\varnothing } m_{1}\left( \xi _{1}\right) m_{2}\left( \xi _{2}\right) . \end{aligned}$$

where $\xi _{1}$and $\xi _{2}$ are focal elements from $M_1$ and $M_2$, respectively. *K* represents the degree of conflict between the pieces of evidence.

## Three-way decision with HFLT expression of loss functions

In the classic 3WD model, it is usually necessary to utilize the information table and the cost matrix to deduce the 3WD decision rules according to the Bayesian minimum cost principle. Many researchers consider that the cost functions of each alternative are identical. In the classic 3WD model, it is usually necessary to utilize the information table and the cost matrix to deduce the 3WD decision rules according to Bayesian minimum cost principle, and many researches considers that the cost functions of each alternative are identical. However, different alternatives usually have different costs when taking the same decision action. For this reason, we consider the related cost functions for each alternative. In this section, we describe the cost function in the 3WD model using HFLTs and deduce the 3WD decision rules. The 3WD model with HFLTs includes a state set $\Omega =\left\{ X,\lnot X \right\}$ and an action set $\mathscr {A}=\left\{ {{a}_{P}},{{a}_{B}},{{a}_{N}} \right\}$ as well. The cost function matrix of the alternatives can then be obtained by expert evaluation of the alternatives under the HFL decision environment. The cost matrix of an alternative is shown in Table 1.

**Table 1:** The cost matrix with HFLTs.

	X	$\lnot X$
$a_P$	$\psi _{PP}^ {h_{S}}$	$\psi _{PN}^ {h_{S}}$
$a_B$	$\psi _{BP}^ {h_{S}}$	$\psi _{BN}^ {h_{S}}$
$a_N$	$\psi _{NP}^ {h_{S}}$	$\psi _{NN}^ {h_{S}}$

In Table 1, we can obtain the cost $\psi _{\vartriangle \vartriangle }^ {h_{S}}$ $(\vartriangle =P,B,N)$ of each decision action for the alternative in different states. Where $\psi _{PP}^ {h_{S}}$, $\psi _{BP}^ {h_{S}}$ and $\psi _{NP}^ {h_{S}}$ represent the cost with HFLE of taking actions $a_P, a_B,a_N$, when the alternative $u_i \in U$ satisfies the condition of state X, respectively. Similarly, $\psi _{PN}^ {h_{S}}$, $\psi _{BN}^ {h_{S}}$ and $\psi _{NN}^ {h_{S}}$ represent the cost with HFLE of taking actions $a_P, a_B, a_N$, when the alternative $u_i \in U$ satisfies the condition of state $\lnot X$, respectively. Meanwhile, according to Definition 3, taking the state set $\Omega =\left\{ X,\lnot X \right\}$ as a non-empty finite universe, we can obtain ${H^{1}_S} = \{ \psi _{PP}^ {h_{S}},\psi _{PN}^ {h_{S}} \}$, ${H^{2}_S} = \{ \psi _{BP}^ {h_{S}},\psi _{BN}^ {h_{S}} \}$, and ${H^{3}_S} = \{ \psi _{NP}^ {h_{S}},\psi _{NN}^ {h_{S}} \}$ three HFLTSs. However, there will be different costs in practical decision-making issues for each alternative $u_i \in U$, so we should consider the cost function for each alternative.

The cost of making the right decision about an alternative in real life is usually less than the wrong and pending decision, then the cost function with HFLE should satisfy the following relation:

13 $$\begin{aligned}{} & {} \psi _{PP}^ {h_{S}} \preceq \psi _{BP}^ {h_{S}} \prec \psi _{NP}^ {h_{S}}. \end{aligned}$$

14 $$\begin{aligned}{} & {} \psi _{NN}^ {h_{S}} \preceq \psi _{BN}^ {h_{S}} \prec \psi _{PN}^ {h_{S}}. \end{aligned}$$

According to Definition 5, we can get the following relation:

15 $$\begin{aligned}{} & {} G(\psi _{PP}^ {h_{S}}) \preceq G(\psi _{BP}^ {h_{S}}) \prec G(\psi _{NP}^ {h_{S}}). \end{aligned}$$

16 $$\begin{aligned}{} & {} G(\psi _{NN}^ {h_{S}}) \preceq G(\psi _{BN}^ {h_{S}}) \prec G(\psi _{PN}^ {h_{S}}). \end{aligned}$$

Then, according to the classic three-way decision process mentioned in Section 2, we can calculate the expected costs of taking different actions for each alternative $u_i$, $u_i\in U$ as follows:

17 $$\begin{aligned}&\mathbb {E}({a}_{P}|u_i)=G(\psi _{PP}^ {h_{S}})Pr(X | u_i)+G(\psi _{PN}^ {h_{S}})Pr(\lnot X | u_i),\nonumber \\&\mathbb {E}({a}_{B}|u_i)=G(\psi _{BP}^ {h_{S}})Pr(X | u_i)+G(\psi _{BN}^ {h_{S}})Pr(\lnot X | u_i),\nonumber \\&\mathbb {E}({a}_{N}|u_i)=G(\psi _{NP}^ {h_{S}})Pr(X | u_i)+G(\psi _{NN}^ {h_{S}})Pr(\lnot X | u_i). \end{aligned}$$

Subsequently, the decision rules are deduced according to the principle of minimum cost:

18 $$\begin{aligned}&\text{(P2) } \ \text{ If } \ \mathbb {E}({a}_{P}|u_i)\le \mathbb {E}({a}_{B}|u_i),\mathbb {E}({a}_{P}|u_i)\le \mathbb {E}({a}_{N}|u_i),\text{ dicide }\ u_i\in POS(X);\nonumber \\&\text{(B2) } \ \text{ If } \ \mathbb {E}({a}_{B}|u_i)\le \mathbb {E}({a}_{P}|u_i),\mathbb {E}({a}_{B}|u_i)\le \mathbb {E}({a}_{N}|u_i),\text{ dicide }\ u_i\in BND(X);\nonumber \\&\text{(N2) } \ \text{ If } \ \mathbb {E}({a}_{N}|u_i)\le \mathbb {E}({a}_{P}|u_i),\mathbb {E}({a}_{N}|u_i)\le \mathbb {E}({a}_{B}|u_i),\text{ dicide }\ u_i\in NEG(X). \end{aligned}$$

Decision rules (P1)-(N1) can be rewritten as (P3)-(N3).

19 $$\begin{aligned}&\text{(P3) } \ \text{ If } \ Pr(X|u_i)\ge \alpha ,\text{ dicide }\ u_i\in POS(X);\nonumber \\&\text{(B3) } \ \text{ If } \ \beta< Pr(X|u_i) < \alpha ,\text{ dicide }\ u_i\in BND(X);\nonumber \\&\text{(N3) } \ \text{ If } \ Pr(X|u_i)\le \beta ,\text{ dicide }\ u_i\in NEG(X). \end{aligned}$$

where

20 $$\begin{aligned} \alpha =\frac{(G(\psi _{PN}^ {h_{S}})-G(\psi _{BN}^ {h_{S}}))}{(G(\psi _{PN}^ {h_{S}})-G(\psi _{BN}^ {h_{S}}))+(G(\psi _{BP}^ {h_{S}})-G(\psi _{PP}^ {h_{S}}))},\nonumber \\ \beta =\frac{(G(\psi _{BN}^ {h_{S}})- (G(\psi _{NN}^ {h_{S}}))}{(G(\psi _{BN}^ {h_{S}})- G(\psi _{NN}^ {h_{S}}))+(G(\psi _{NP}^ {h_{S}})- G(\psi _{BP}^ {h_{S}}))}. \end{aligned}$$

## The estimation of conditional probability

In Section 3, we describe the determination of decision rules for a 3WD model with multiple cost functions under the HFL environment. At the same time, conditional probability calculation is also an essential issue in the decision-making procedure of the 3WD model^40^. In this section, we first discuss the construction of the evidence matrix. Subsequently, we discuss the computation of conditional probabilities utilizing methods based on DSET and TOPSIS.

### Construction of evidence matrixes based on the information system with HFL

In the decision-making process of 3WD, the calculation of the expected cost of taking the corresponding decision action for each alternative $u_i \in U$ and the derivation of the decision rule requires the support of the conditional probability $Pr(X|u_i)$, as shown in Eqs. (17)–(19). Generally, the conditional probability was computed employing information tables and equivalence classes divided by equivalence relations. Nevertheless, in real situations, the equivalence relation cannot be obtained directly and the information loss will be caused by fusing multiple information tables through the aggregation operator in the group decision environment. Therefore, we utilize the DSET-based TOPSIS method to calculate conditional probabilities.

Let $U = \{u_1,u_2,\cdots ,u_m\}$ be denoted as the set of alternatives to be evaluated and each alternative is assessed by a set of experts $E = \{e_1, e_2,\cdots ,e_t\}$. $P = \{p_1,p_2,\cdots ,p_n\}$ represents the attribute set of the HFL information systems. The weight vector is defined as : $W =\{w_1,w_2,\cdots ,w_n\}^T$, it meets the relations: $0 \le w_j \le 1$ and $\sum _{j=1}^n w_j = 1$. Let $S = \{ {s}_{\gamma } | \gamma = \ \text {-}h,...,0,...,h \}$ be an LTS, which also serves as the frame of discernment in DSET. In the evaluation problem of the alternative $u_i$, according to Definition 2, we make the attribute set *P* as a finite and non-empty universe, and the evaluation of $u_i$ regarding the attribute $p_j$ as an HFLE defined in *S*. At the same time, it is also a proposition in *S*, and all HFLEs on alternative $u_i$ constitute an HFLTS. The evaluation of the alternative $u_i$ regarding the attribute $p_j$ by expert $e_k$ is denoted as $d_{ij}^{k}$, and the evaluation of all alternatives by expert $e_k$ is expressed in a hesitant fuzzy linguistic decision matrix $D^{k} = [d_{ij}^{k}]_{m\times n}$. The decision matrix is shown below:

$$\begin{aligned} D^{k} = \begin{pmatrix} d_{11}^{k},&{}d_{12}^{k},&{}\dots ,&{}d_{1n}^{k}\\ d_{21}^{k},&{}d_{22}^{k},&{}\dots ,&{}d_{2n}^{k}\\ \vdots &{}\vdots &{}\ddots &{} \vdots \\ d_{m1}^{k},&{}d_{m2}^{k},&{}\dots ,&{}d_{mn}^{k} \end{pmatrix} \end{aligned}$$

In the decision matrix, experts describe the evaluation results using linguistic expressions, thus we can transform linguistic expressions into HFLEs by Definition 6. After translation, the evaluation of the alternative $u_i$ regarding the attribute $p_j$ by expert $e_k$ is denoted as $\tilde{d}_{ij}^{k}$, and the decision matrix is denoted as $\tilde{D}^k = [\tilde{d}_{ij}^{k}]_{m\times n}$. We apply the belief matrix computation method proposed by Liu et al.^34^ in the context of double hesitant fuzzy linguistic environment to the HFL environment, while making certain additional enhancements.Then we calculate the evidence group decision matrix by the following steps.

- First we need to calculate the distance between two elements $\tilde{d}_{ij}^{k}$ and $\tilde{d}_{ij}^{r}$. Let $S = \{ {s}_{\gamma } | \gamma =$-$h,...,0,...,h \}$ be a linguistic term set and $\tilde{d}_{ij}^{r}$ , $\tilde{d}_{ij}^{k}= \{s_{\gamma _{l}} \mid s_{\gamma _{l}} \in S, l=1,2, \cdots , L$ } are an HFLE, they denoted the evaluation of the alternative $u_i$ regarding the attribute $p_j$ by experts $e_r$ and $e_k$, respectively. The distance between two HFLEs can be calculated using Hamming distance:
  21 $$\begin{aligned} H\left( \tilde{d}_{ij}^{k}, \tilde{d}_{ij}^{r} \right) = \frac{1}{L}\sum _{l=1}^L |g_{ij}^k(\gamma _{l}) - g_{ij}^r(\gamma _{l}) |. \end{aligned}$$
  where $g_{ij}^k(\gamma _{l}), g_{ij}^r(\gamma _{l})$ denoted that the transformation function.

#### Remark 1

Note that the lengths ${L}_{ij}^{k},{L}_{ij}^{r}$ of the two HFLEs $\tilde{d}_{ij}^{k}, \tilde{d}_{ij}^{r}$ may be different, this will cause the Hamming distance to not be calculated properly. Assumption ${L}_{ij}^{k}>{L}_{ij}^{r}$, there will be ${L}_{ij}^{k} - {L}_{ij}^{r}$ missing values in $\tilde{d}_{ij}^{r}$, then we fill in the missing values by calculating the mean value *m* by the following formula:

22 $$\begin{aligned} m = \frac{1}{{L_{ij}^{r}}}\sum _{l=1}^{L_{ij}^{r}} \ \gamma _{l}. \end{aligned}$$

- Calculate the similarity degree between the HFLEs $\tilde{d}_{ij}^{k}, \tilde{d}_{ij}^{r}$ by
  23 $$\begin{aligned} sim\left( \tilde{d}_{ij}^{k}, \tilde{d}_{ij}^{r}\right) = 1 - \frac{H\left( \tilde{d}_{ij}^{k}, \tilde{d}_{ij}^{r} \right) }{2h}. \end{aligned}$$
  where $sim\left( \tilde{d}_{ij}^{k}, \tilde{d}_{ij}^{r}\right)$ represents the evaluated similarity degree of the alternative $u_i$ regarding the attribute $p_j$ by expert $e_k$ and $e_r$.
- Calculate the support degree between two HFLEs of the experts.
  24 $$\begin{aligned} sup\left( \tilde{d}_{ij}^{k}\right) = \sum _{r=1,r \ne k}^t sim\left( \tilde{d}_{ij}^{k}, \tilde{d}_{ij}^{r}\right) . \end{aligned}$$
  where t denotes the total number of experts in the set *E*.
- Calculate the belief degree.
  25 $$\begin{aligned} bel\left( \tilde{d}_{ij}^{k}\right) = \frac{sup\left( \tilde{d}_{ij}^{k}\right) }{\sum _{k=1}^t sup\left( \tilde{d}_{ij}^{k}\right) }. \end{aligned}$$
- Generate a group decision matrix based on the evidence. In the light of Liu et al.^58^, LTS $S = \{ {s}_{\gamma } | \gamma = \ \text {-}h,...,0,...,h \}$ able to act as a FOD, let $\tilde{d}_{ij}^{k}(k = 1,2,...t)$ denotes all experts’ evaluations of alternative $u_i$ with respect to attribute $p_j$. A focal element set $F_{ij} = \{\tilde{d}_{ij}^{k} |k = 1, 2,...,t \}$ over alternative $u_i$ on attribute $p_j$ is composed of all $\tilde{d}_{ij}^{k}$ together. And the BPA values of a focal element can be derived from the belief degrees of experts^34^. Then, the belief sets of different experts over alternative $u_i$ on attribute $p_j$ can be constructed, denoted by $\tilde{d}_{ij} =\left\{ \left( \tilde{d}_{ij}^{k}, bel\left( \tilde{d}_{ij}^{k}\right) \right) |k = 1, 2, . . . ,t \right\} (i = 1, 2, . . . , m; j =1, 2, . . . , n)$, and the evidence group decision matrix can be denoted by:
  26 $$\begin{aligned} D = \left( \tilde{d}_{ij} \right) _{m \times n}. \end{aligned}$$

### Extended TOPSIS method based on DSET for HFLEs

In Section 4.1, we discuss the determination of the group decision matrix based on DSET under the HFL environment. The TOPSIS method is extended to group decision matrices based on evidence in the following and discuss the computation of conditional probabilities in the HFL information system.

The process of the TOPSIS method is to calculate the positive and negative ideal solutions in the information table separately and sort the alternatives according to their distance from the positive and negative ideal solutions. We can calculate the expected value of the $\tilde{d}_{ij}$ over alternative $u_i$ on attribute $p_j$ according to the expected function of the $\tilde{d}_{ij}$ as follows:

27 $$\begin{aligned} Q_{ij}= & {} \frac{bel\left( \tilde{d}_{ij}^{k}\right) }{t}\sum _{l=1}^{t} g\left( \gamma _l\right) . \end{aligned}$$

28 $$\begin{aligned} e_{ij}= & {} \sum _{i=1}^{|\tilde{d}_{ij}|} Q_{ij}. \end{aligned}$$

where $|\tilde{d}_{ij}|$ represents the cardinality of the set $\tilde{d}_{ij}$.

Then, we can obtain the evidence expected value group decision matrix denoted by:

29 $$\begin{aligned} E = \left( e_{ij}\right) _{m \times n}. \end{aligned}$$

According to the general process of the TOPSIS method, we calculate the conditional probability through the following steps:

- The conversion of reverse indicators. In the decision matrix, the decision attributes include benefit type and cost type, and the two types of attributes have different evaluation scales. Therefore, we need to converse the attribute value of cost type. Let $e_{ic}$ represents the evaluation value of the alternative *i* in the cost attribute *c*, $max\left( e_{c}\right)$ represents the maximum value of all alternatives under attribute c, and the conversion formula is as follows:
  30 $$\begin{aligned} e_{ic} = max\left( e_{c}\right) - e_{ic}\left( i = 1,2,...,m\right) . \end{aligned}$$
  where *m* is the number of alternatives.
- Above all, we calculate the positive ideal solution set $x_{i}^+ = (e_{1}^{+,i}, e_{2}^{+,i}, \cdots ,e_{n}^{+,i} )$, and the negative ideal solution set $x_{i}^- = (e_{1}^{-,i}, e_{2}^{-,i}, \cdots ,e_{n}^{-,i} )$, according to the evidence expected value group decision matrix, where
  31 $$\begin{aligned} x_{j}^{+}= & {} \max _{1<i<m }\{e_{ij}\}. \end{aligned}$$
  32 $$\begin{aligned} x_{j}^{-}= & {} \min _{1<i<m }\{e_{ij}\}. \end{aligned}$$
- After that, the distances $d_{i}^+$ and $d_{i}^-$ between the alternative $u_i \in U$ and the positive and negative ideal solutions are calculated by the following equations, respectively:
  33 $$\begin{aligned} d_{i}^+= & {} \sqrt{\sum _{j=1}^n w_j\left( x_{j}^{+}- e_{ij} \right) ^2}. \end{aligned}$$
  34 $$\begin{aligned} d_{i}^-= & {} \sqrt{\sum _{j=1}^n w_j\left( x_{j}^{-} - e_{ij} \right) ^2}. \end{aligned}$$
- At last, according to the positive and negative ideal solutions of the alternative $u_i \in U$, the comprehensive score value of $u_i$ can be calculated as follows:
  35 $$\begin{aligned} RC_i= \frac{d_{i}^-}{d_{i}^+ + d_{i}^-}. \end{aligned}$$
  Due to the effectiveness of the TOPSIS method in MADM, Liang et al.^42^ and Wang et al.^40^ argue that the comprehensive score value can be regarded as the conditional probability $Pr(X | u_i)$ that the alternatives belong to *X*. This is due to the ability to use the TOPSIS method to calculate scores for alternatives in the information table, which typically reflect the similarity of each decision option relative to the ideal solution. These scores can be used as a representation of conditional probabilities because they provide the likelihood that each alternative will be selected as the best decision. The greater the likelihood, the greater the probability of being in the positive region. Therefore, for each alternative $u_i \in U$, the conditional probability $Pr(X | u_i) = RC_i$.

## The decision-making steps and the algorithm

In light of the the description of the various processes of decision-making above, the key steps of our proposed 3WD model based on DSET are described as follows:

**Step 1:** Transform the linguistic expression result matrices into decision tables according to the transformation functions $T_{G_H}$ in Section 4.1.
**Step 2:** Calculate the Hamming distance, the similarity degree, the support degree, and the belief degree by Eqs. (21), (23), (24) and (25), respectively.
**Step 3:** Obtain the BPA values of the focal elements in the decision tables according to Definition 6 and Step 5 in Section 4.1.
**Step 4:** Construct the evidence group decision matrix $D = \left( \tilde{d}_{ij} \right) _{m \times n}$ with the aid of the focus elements and their BPA values.
**Step 5:** Calculate the expected values of the evidence by equations Eqs. (27) and (28).
**Step 6:** Determine the attribute type and convert the cost attribute using Eq. (30). After that, the conditional probability of each alternative is calculated based on the expected values matrix and Eqs. (31)–(35).
**Step 7:** Calculate thresholds $\alpha$ and $\beta$ for each alternative using Eq. (20) and the cost matrices.
**Step 8:** Divide alternatives into three regions with the aid of decision rules (P3) - (N3).

Subsequently, the specific description of the proposed 3WD model based on DSET is shown in Algorithm 1.

### Remark 2

In Algorithm 1, the time complexity of Step 1 and Step 6 is O(mn), the time complexity of Step 2, Step 4, and Step 5 is $O(n^2 m)$, and the time complexity of Step 3, Step 7, and Step 8 is O(m), so the time complexity of Algorithm 1 is $O(n^2 m)$.

**Algorithm 1:**
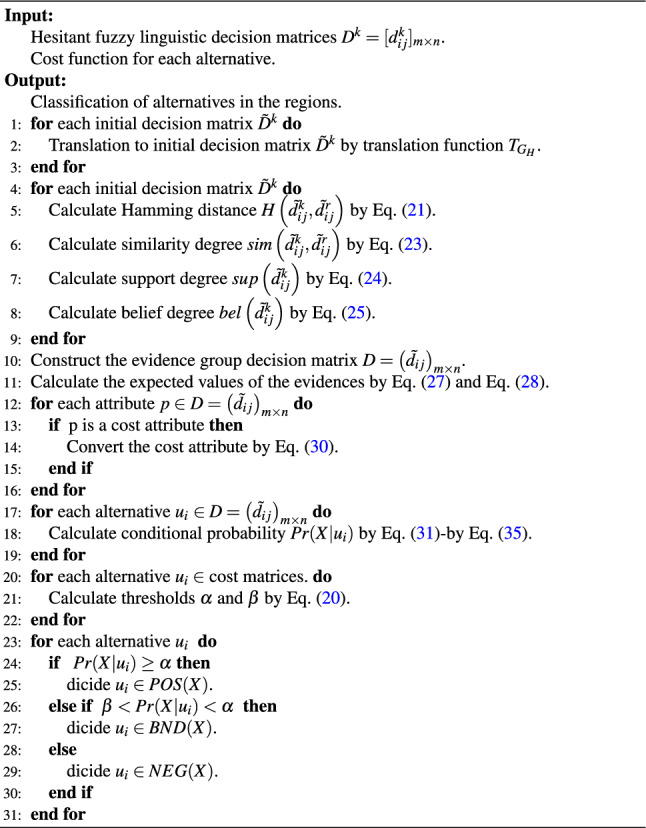
The algorithm of the proposed 3WD model

## Illustrative example

In this section, we utilize a case study on postgraduate course evaluation to illustrate the decision process of our proposed 3WD model, which is a case of the MAGDM issue adapted from Liu et al.^34^

### Problem description

Let $U = \{ u_1,u_2,u_3,u_4\}$ be four courses to be evaluated, *P* = {*Practicability* $(p_1)$, *Completion of task* $(p_2)$, *Effectiveness* $(p_3)$, *Attraction* $(p_4)$ } be four evaluation criteria for assessing the quality of postgraduate courses. In addition, all the course quality assessment results are expressed in linguistic terms by four experts $E = \{e_1, e_2, e_3 ,e_4\}$.

In this issue, each course has two states $\Omega =\left\{ X,\lnot X \right\}$ representing the assessment of course quality as qualified or unqualified, respectively. Furthermore, each course has action set $\mathscr {A}=\left\{ {{a}_{P}},{{a}_{B}},{{a}_{N}} \right\}$, which represents the decision actions of accepting, delaying, and rejecting, respectively. In addition, experts expresses the evaluation in the form of linguistic expressions based on a LTS $S = \{ s_{\text {-}3}, s_{\text {-}2},s_{\text {-}1},s_{0}, s_{1}, s_{2}, s_{3}\}$ = {*very bad*,*bad*, *a little bad*, * medium *, *a little good *, *good *, *very good *}, and the original results of the expert evaluation are shown in Tables 2, 3 and 4. In this case, we let the weight matrix corresponding to the criteria set *P* to be $W = \{0.45, 0.2, 0.25, 0.1\}^T$, and the cost matrix is shown in Table 5.

**Table 2:** Course evaluation results provided by expert 1.

	$p_1$	$p_2$	$p_3$	$p_4$
$u_1$	Between medium and a little good	Medium	Between a little bad and medium	Between a little bad and medium
$u_2$	Medium	Between a little bad and medium	A little bad	Between a little bad and medium
$u_3$	Between good and very good	A little bad	Between a little good and good	Good
$u_4$	A little bad	Medium	Bad	Between a little bad and medium

**Table 3:** Course evaluation results provided by expert 2.

	$p_1$	$p_2$	$p_3$	$p_4$
$u_1$	A little bad	Medium	Between a little good and good	Between very bad and bad
$u_2$	A little bad	Between medium and a little good	Medium	Between bad and a little bad
$u_3$	Between medium and a little good	A little bad	Bad	Between a little bad and medium
$u_4$	Between a little bad and medium	Medium	A little good	Between medium and a little good

**Table 4:** Course evaluation results provided by expert 3.

	$p_1$	$p_2$	$p_3$	$p_4$
$u_1$	Good	Very good	A little bad	Between medium and a little good
$u_2$	Between medium and a little good	Good	Between a little bad and medium	Between bad and a little bad
$u_3$	Good	Between medium and a little Good	Medium	Between good and very good
$u_4$	Between medium and a little good	Good	Between a little bad and medium	A little good

**Table 5:** Cost matrices of four courses.

	*PP*	*BP*	*NP*	*PN*	*BN*	*NN*
$u_1$	$\{ s_\text {-3}\}$	$\{ s_\text {-1},s_0\}$	$\{ s_3\}$	$\{ s_2,s_3\}$	$\{ s_\text {-1}\}$	$\{ s_\text {-3}\}$
$u_2$	$\{ s_\text {-3}, s_\text {-2}\}$	$\{ s_\text {-1}\}$	$\{ s_2,s_3\}$	$\{ s_2,s_3\}$	$\{ s_0,s_1\}$	$\{ s_\text {-2}\}$
$u_3$	$\{ s_\text {-2}\}$	$\{ s_0,s_1\}$	$\{ s_2,s_3\}$	$\{ s_3\}$	$\{ s_0\}$	$\{ s_\text {-2}\}$
$u_4$	$\{ s_\text {-3}, s_\text {-2}\}$	$\{ s_\text {-1},s_0\}$	$\{ s_3\}$	$\{ s_2,s_3\}$	$\{ s_0\}$	$\{ s_\text {-3}, s_\text {-2}\}$

### Decision process based on the proposed model

With help from the proposed DSET-based 3WD method, in this subsection, we describe the decision-making process for the course quality assessment issue, and the specific steps are shown below:

**Step 1:** For all three linguistic expression results matrices of experts, we can transform the linguistic expression into three individual decision tables $\widetilde{d}_{ij}^k (k = 1,2,3)$ with HFLEs through the transformation function $T_{G_H}$ in Section 4.1, as shown in Tables 6, 7 and 8.

**Table 6:** Initial decision matrix of expert 1.

	$p_1$	$p_2$	$p_3$	$p_4$
$u_1$	$\{s_0, s_1\}$	$\{ s_0 \}$	$\{s_\text {-1}, s_0\}$	$\{s_\text {-1}, s_0\}$
$u_2$	$\{ s_0\}$	$\{s_\text {-1}, s_0\}$	$\{s_\text {-1} \}$	$\{s_\text {-1}, s_0\}$
$u_3$	$\{s_2, s_3\}$	$\{s_\text {-1} \}$	$\{s_1, s_2\}$	$\{ s_2\}$
$u_4$	$\{s_\text {-1} \}$	$\{ s_0\}$	$\{s_\text {-2}\}$	$\{s_\text {-1}, s_0\}$

**Table 7:** Initial decision matrix of expert 2.

	$p_1$	$p_2$	$p_3$	$p_4$
$u_1$	$\{ s_\text {-1} \}$	$\{ s_0 \}$	$\{ s_1,s_2 \}$	$\{s_\text {-3}, s_\text {-2} \}$
$u_2$	$\{ s_\text {-1} \}$	$\{ s_0,s_1\}$	$\{ s_0 \}$	$\{s_\text {-2}, s_\text {-1} \}$
$u_3$	$\{ s_0,s_1 \}$	$\{ s_\text {-1} \}$	$\{s_\text {-2} \}$	$\{s_\text {-1}, s_0 \}$
$u_4$	$\{s_\text {-1}, s_0 \}$	$\{ s_0 \}$	$\{ s_1 \}$	$\{ s_0,s_1\}$

**Table 8:** Initial decision matrix of expert 3.

	$p_1$	$p_2$	$p_3$	$p_4$
$u_1$	$\{ s_2 \}$	$\{ s_3 \}$	$\{s_\text {-1} \}$	$\{ s_0,s_1\}$
$u_2$	$\{ s_0,s_1\}$	$\{ s_2 \}$	$\{s_\text {-1}, s_0 \}$	$\{s_\text {-2}, s_\text {-1} \}$
$u_3$	$\{ s_2 \}$	$\{ s_0,s_1\}$	$\{ s_0 \}$	$\{ s_2,s_3 \}$
$u_4$	$\{ s_0,s_1\}$	$\{ s_2 \}$	$\{s_\text {-1}, s_0 \}$	$\{s_1\}$

**Step 2:** Based on Tables 6, 7, and 8, we can calculate the Hamming distance, the similarity degree, the support degree, and the belief degree by Eqs. (21), (23), (24) and (25), respectively.

**Step 3:** According to Definition 7 and Step 5 in Section 4.1, we can obtain the BPA values for the focal elements in information tables.

**Step 4:** Evidence group decision matrix $D = \left( \tilde{d}_{ij} \right) _{m \times n}$, which is shown in Tables 9 and 10, can be constructed from focal elements and their BPA values.

**Table 9:** Evidence group decision matrix with attributes $p_1$, $p_2$.

	$p_1$	$p_2$
$u_1$	{ ( $\{ s_0, s_1 \}$, 0.3382 ), ( $\{ s_\text {-1} \}$, 0.3309) , ( $\{ s_2 \}$, 0.3309) }	{ ($\{ s_0 \}$, 0.6764 ), ($\{ s_3 \}$ , 0.3235 ) }
$u_2$	{ ( $\{ s_0\}$ , 0.3357 ), ($\{ s_\text {-1} \}$,0.3310) , ($\{ s_0,s_1\}$, 0.3333 ) }	{ ( $\{s_\text {-1}, s_0 \}$, 0.3325 ), ( $\{ s_0,s_1\}$, 0.3374), ( $\{ s_2 \}$ , 0.3301) }
$u_3$	{ ($\{s_2, s_3\}$, 0.3341 ), ($\{ s_0,s_1 \}$ ,0.3293 ), ($\{ s_2 \}$ , 0.3365 ) }	{ ($\{ s_\text {-1} \}$ , 0.6714), ($\{ s_0,s_1\}$, 0.3286 ) }
$u_4$	{ ($\{s_\text {-1} \}$, 0.3333 ), ($\{s_\text {-1}, s_0 \}$,0.3357 ), ( $\{ s_0,s_1\}$, 0.3310) }	{ ( $\{ s_0 \}$, 0.673 ), ($\{ s_2\}$, 0.3269) }

**Table 10:** Evidence group decision matrix with attributes$p_3$, $p_4$.

	$p_3$	$p_4$
$u_1$	{ ( $\{s_\text {-1}, s_0\}$, 0.3374), ( $\{ s_1,s_2 \}$ , 0.3277 ) ,($\{s_\text {-1} \}$ , 0.3350)	{ ($\{s_\text {-1}, s_0\}$ , 0.3382), ( $\{s_\text {-3}, s_\text {-2} \}$ , 0.3284), ($\{ s_0,s_1\}$ , 0.3333 )
$u_2$	{ ( $\{s_\text {-1} \}$ , 0.3325) , ($\{ s_0 \}$, 0.3325) , ($\{s_\text {-1}, s_0 \}$, 0.3349) }	{ ($\{s_\text {-1}, s_0\}$, 0.3301), ($\{s_\text {-2}, s_\text {-1} \}$ ,0.6698 ) }
$u_3$	{ ($\{s_1, s_2\}$, 0.3317 ), ($\{s_\text {-2} \}$, 0.3292), ($\{ s_0 \}$, 0.3391 ) }	{ ( $\{ s_2\}$ , 0.3382 ), ( $\{s_\text {-1}, s_0 \}$ ,0.3260 ),( $\{ s_2,s_3 \}$ , 0.3358) }
$u_4$	{ ( $\{s_\text {-2}\}$, 0.3309), ($\{ s_1 \}$, 0.3309 ) ,( $\{s_\text {-1}, s_0 \}$, 0.3382) }	{ ($\{s_\text {-1}, s_0\}$,0.3310 ), ($\{ s_0,s_1\}$,0.3357 ), ($\{s_1\}$ , 0.3333 ) }

**Step 5:** Based on the evidence group decision matrix, the evidence obtained can be used to calculate its expected value by Eqs. (27) and (28), which is shown in Table 11.

**Table 11:** Expected values of the evidence in the matrix.

	$p_1$	$p_2$	$p_3$	$p_4$
$u_1$	0.5833	0.6618	0.498	0.3627
$u_2$	0.4726	0.6104	0.4167	0.305
$u_3$	0.7788	0.4155	0.4732	0.7255
$u_4$	0.444	0.609	0.4167	0.556

**Step 6:** In this case, all attributes are benefit type attributes, thus we don’t need to convert using Eq. (30). Based on the expected values matrix, the conditional probability of each alternative can be calculated by Eqs. (31)–(35) and the results are displayed in Table 12.

**Table 12:** Conditional probability outcome values for alternatives.

	$u_1$	$u_2$	$u_3$	$u_4$
Conditional probability	0.4219	0.2402	0.7264	0.3696

**Step 7:** Based on Table 5, we collect the HFLEs cost evaluation results for each alternatives. Then, Eq. (20) can be used to calculate thresholds $\alpha$ and $\beta$ for each alternative, which is shown in Table 13.

**Table 13:** Thresholds $\alpha$ and $\beta$ for each alternative.

	$u_1$	$u_2$	$u_3$	$u_4$
$\alpha$	0.583	0.571	0.545	0.556
$\beta$	0.364	0.417	0.5	0.417

**Step 8:** In light of the decision rules (P3) - (N3), alternatives can be divided into three decision regions i.e. POS(X) = $\{ u_3 \}$, BND(X) = $\{ u_1 \}$, NEG(X) = $\{ u_2, u_4\}$. Therefore, the decision results of the proposed model can be interpreted as: after combining the assessments of three experts, the course quality evaluation of $u_3$ is qualified, and $u_2$ and $u_4$ are unqualified. Meanwhile, $u_1$ needs to make further judgments based on more information.

## Comparative analysis

In this section, we will illustrate the effectiveness and practicability of our proposed model through comparative analysis in four cases.

### Comparative analysis with the method for MAGDM in the DSET framework

First of all, we compare our model with a MAGDM method^58^ in the DSET framework and this method can handle the MAGDM problem with HFL information. In this comparison experiment, we mainly verify the effectiveness of the model’s fusion method under hesitant fuzzy linguistic environment, and compare the differences in fusion results and the differences in the classification of alternatives between three-way decision-making and two-way decision-making. Therefore, we employ the case study conducted by Liu et al.^58^ on green supplier selection for the purpose of comparative analysis.

In this question, six green suppliers $U = \{ u_1,u_2,u_3,u_4,u_5,u_6 \}$ are evaluated by four experts $E = \{e_1, e_2, e_3 ,e_4\}$, and each supplier contains five evaluated attributes *P* = { $p_1$, $p_2$, $p_3$,$p_4$, $p_5$}. In addition, we use the same attribute weight vector $W = \{0.2, 0.15, 0.2, 0.2, 0.25\}^T$ and linguistic term sets. Subsequently, the conditional probabilities of each alternative can be obtained after the calculation of our proposed model as shown in Table 14.

**Table 14:** Conditional probability outcome values for alternatives.

	$u_1$	$u_2$	$u_3$	$u_4$	$u_5$	$u_6$
Conditional probability	0.6271	0.3810	0.7536	0.5624	0.4345	0.7028

Based on the research conducted by Liu et al., a DSET-based two-way decision-making method is used, and the decision-making result is represented by a ranking relation $u_3 \succ u_6 \succ u_1 \succ u_4 \succ u_5 \succ u_2$. However, this decision-making method does not consider the cost function of each alternative, suppliers can only be selected according to the ranking result. The TOPSIS method used in this paper can not only be used as a calculation method of conditional probability but can rank the alternatives. Therefore, we can convert the conditional probability to the ranking relationship according to the size of the value for comparative analysis, and the conversion result is $u_3 \succ u_6 \succ u_1 \succ u_4 \succ u_5 \succ u_2$.

In light of the ranking results, it can be learned that our method is the same as the ranking results of Liu et al. for suppliers. Further analysis, according to Table 15, it can be found that our model calculates a larger span of score values between alternatives, while the span of score values in the literature^58^ is smaller, which can indicate the fusion of the models in this chapter. Computational methods have better discrimination between alternatives. In addition, corresponding to the three-way decision-making model, if the thresholds are the same, alternatives can be better classified with higher discrimination. In our model, the conditional probability is calculated by the TOPSIS method, which can reflect the superiority of the alternative over other alternatives, and therefore can be analyzed as the comprehensive score of the alternative.

**Table 15:** Comparison of the score values of six alternatives.

	$u_1$	$u_2$	$u_3$	$u_4$	$u_5$	$u_6$
Our model	0.6271	0.3810	0.7536	0.5624	0.4345	0.7028
The model of Liu et al.^58^	0.659	0.535	0.677	0.662	0.570	0.667

In addition, the cost of taking different actions for suppliers is considered in our model. Therefore, we will compare the impact on ranking results when considering the cost of action. Assuming that the supplier cost functions given by the experts is shown in Table 16. In this case three suppliers need to be selected, so we can transform the ranking relationship into the the positive region POS(X) = $\{u_1,u_3,u_6\}$ and the negative region NEG(X) = $\{u_2,u_4,u_5\}$. From Table 17, we can learn that the decision result after adding the cost functions is different from the decision result in reference^58^. The difference according to our results is that, supplier $u_4$ is classified into *POS*(*X*) from *NEG*(*X*), and supplier $u_6$ is classified into *BND*(*X*) from *POS*(*X*). The reasons for this can be summarized as follows.

**Table 16:** Cost functions of six suppliers.

	*PP*	*BP*	*NP*	*PN*	*BN*	*NN*
$u_1$	$\{ s_\text {-3}\}$	$\{ s_\text {-1},s_0\}$	$\{ s_3\}$	$\{ s_2,s_3\}$	$\{ s_\text {-1}\}$	$\{ s_\text {-3}\}$
$u_2$	$\{ s_\text {-3}, s_\text {-2}\}$	$\{ s_\text {-1}\}$	$\{ s_2,s_3\}$	$\{ s_2,s_3\}$	$\{ s_0,s_1\}$	$\{ s_\text {-2}\}$
$u_3$	$\{ s_\text {-2}\}$	$\{ s_0,s_1\}$	$\{ s_2,s_3\}$	$\{ s_3\}$	$\{ s_0\}$	$\{ s_\text {-2}\}$
$u_4$	$\{ s_\text {-3}, s_\text {-2}\}$	$\{ s_\text {-1},s_0\}$	$\{ s_3\}$	$\{ s_2,s_3\}$	$\{ s_0\}$	$\{ s_\text {-3}, s_\text {-2}\}$
$u_5$	$\{ s_\text {-2}, s_\text {-1}\}$	$\{ s_0\}$	$\{ s_1,s_2\}$	$\{ s_2\}$	$\{ s_\text {-1}\}$	$\{ s_\text {-3} \}$
$u_6$	$\{ s_\text {-3}\}$	$\{ s_\text {-2}\}$	$\{ s_2\}$	$\{ s_3\}$	$\{ s_0\}$	$\{ s_\text {-2}, s_\text {-1}\}$

**Table 17:** The comparison of decision results.

Methods	*POS*(*X*)	*BND*(*X*)	*NEG*(*X*)
Method in reference^58^	$\{u_1,u_3,u_6\}$	$\varnothing$	$\{u_2,u_4,u_5\}$
Our method	$\{u_1,u_3,u_4\}$	$\{u_6\}$	$\{u_2,u_5\}$

- Our proposed approach takes into account the decision cost of each alternative and makes decisions on the basis of the principle of minimum cost. However, the cost caused by decision-making action is not considered in the method of Liu et al.^58^. This will lead to differences in decision-making.
- In our method, we use evidence-based TOPSIS method to analyze the distance between the alternatives and the optimal and worst ideal solutions in the information table, thus, the alternatives can be calculated based on the same standard, and our results can better reflect the difference between the alternative’s relative relationship. When the TOPSIS method is not used, the decision results of alternatives are presented in Table 18. According to the comparison results, we find that the method without TOPSIS classifies $u_2$ into the *BND*(*X*), because this method can not obtain more information to make decisions on $u_2$. Therefore, our method can more accurately reflect the gap between the alternatives, which leads to more reasonable decision results.

**Table 18:** The comparison of decision results without TOPSIS.

Method in reference^58^	$\{u_1,u_3,u_6\}$	$\varnothing$	$\{u_2,u_4,u_5\}$
Our method	$\{u_1,u_3,u_4\}$	$\{u_6\}$	$\{u_2,u_5\}$
Our method(without TOPSIS)	$\{u_1,u_3,u_4\}$	$\{u_2,u_6\}$	$\{u_5\}$

### Comparative analysis of several models for HFLTSs with possibility distributions

In the multiple attribute group decision-making, the decision-making based on the probability distribution of the hesitant fuzzy linguistic term sets is also a popular decision-making method. For example, in reference^59^, the distance measures of the possibility distribution were used to calculate the consensus degree, and two decision models are proposed respectively based on the VIKOR method and the TOPSIS method. In reference^60^, some aggregation operators were proposed based on the probability distribution to calculate the degree of consensus among experts, and a decision model was proposed. These models are also two-way decision-making methods, so we rank the alternatives by the conditional probability values calculated by the TOPSIS method in the proposed method. This method can turn our proposed method into a two-way decision-making method. However, This would not account for the loss of alternatives by taking different actions. In order to further illustrate the effectiveness and adaptability of our model in fusion-calculating conditional probabilities in different cases, we will compare with these models. In the following, we will use the cases in these two papers for comparative analysis.

A case of Health-care waste management is given in reference^59^. Four alternative treatment methods of medical waste $U = \{ u_1,u_2,u_3,u_4 \}$ are evaluated by five experts $E = \{e_1, e_2, e_3 ,e_4,e_5\}$. Each treatment method has six evaluation criteria *P* = { $p_1$, $p_2$, $p_3$,$p_4$, $p_5$, $p_6$} and the weight vector is $W = (0.1875, 0.1587, 0.2027, 0.1528, 0.1741, 0.1242)$. The expert decision matrices with hesitant fuzzy linguistic expression are shown in Tables 1–5 in reference^59^, and the linguistic term set also has seven linguistic terms.

After calculation by our model, the conditional probability values of each alternative, in this case, are shown in Table 19. Therefore, according to the conditional probability values, the priority relationship of all alternatives can be obtained as $u_2 \succ u_3 \succ u_4 \succ u_1$. The ranking results of our model compared with the model based on the VIKOR method and the model based on the TOPSIS method in reference^59^ are shown in Tables 20 and 21, respectively.

**Table 19:** Conditional probability outcome values for alternatives.

	$u_1$	$u_2$	$u_3$	$u_4$
Conditional probability	0.45796	0.67507	0.59237	0.48285

**Table 20:** Ranking comparison with model based on VIKOR.

Model	Case 1	Case 2
VIKOR based model in reference^59^	$u_2 \succ u_3 \succ u_1 \succ u_4$	$u_2 \succ u_3 \succ u_1 \succ u_4$
	$u_2 \succ u_3 \succ u_4 \succ u_1$	$u_2 \succ u_3 \succ u_4 \succ u_1$
	$u_2 \succ u_3 \succ u_1 \succ u_4$	$u_2 \succ u_3 \succ u_4 \succ u_1$
Our method	$u_2 \succ u_3 \succ u_4 \succ u_1$

**Table 21:** Ranking comparison with model based on TOPSIS.

Model	Ranking order
TOPSIS based model in reference^59^	$u_2 \succ u_3 \succ u_1 \succ u_4$
	$$u_2 \succ u_3 \succ u_1 \succ u_4$$
	$$u_2 \succ u_3 \succ u_4 \succ u_1$$
	$$u_2 \succ u_3 \succ u_1 \succ u_4$$
Our method	$u_2 \succ u_3 \succ u_4 \succ u_1$

It can be found in Table 19 that the ranking results of our method are the same as the model based on the VIKOR method in reference^59^ in many cases. It can be found in Table 20 that our method and the TOPSIS-based model pair rank the top two in all cases in total agreement, and the overall ranking is the same in one case. In reference^59^, the best alternative to alternatives for both models is $u_2$, which is consistent with our results. This can illustrate that our method is effective in the problem of optimal alternative selection.

In the following, we will compare the models in In reference^60^. Similarly, we use the case in reference^60^ for comparative analysis. This case is about a personnel evaluation and selection problem. Four candidates $U = \{ u_1,u_2,u_3,u_4 \}$ are evaluated by four experts $E = \{e_1, e_2, e_3,e_4\}$, and the performance of the candidates is assessed through six criteria *P* = { $p_1$, $p_2$, $p_3$,$p_4$, $p_5$, $p_6$}. Similarly, the linguistic term set is composed of seven linguistic terms.

The model in reference^60^ is also a two-way decision model, so we need to use the conditional probability value to convert it into a two-way decision.The model in reference^60^ is also a two-way decision model, so we need to use the conditional probability value to convert it into a two-way decision. The calculation results of the conditional probability are shown in Table 22. According to the conditional probability value of the alternatives, the priority relationship of the alternatives can be obtained as $u_2 \succ u_3 \succ u_4 \succ u_1$.

**Table 22:** Conditional probability outcome values for alternatives.

	$u_1$	$u_2$	$u_3$	$u_4$
Conditional probability	0.43889	0.549976	0.451824	0.443458

**Table 23:** Comparison with results from a decision model with a possibility distribution.

Model	Ranking order
model in^60^	$u_2 \succ u_3 \succ u_4 \succ u_1$
Our method	$u_2 \succ u_3 \succ u_4 \succ u_1$

In this case, the ranking results of our method and the alternatives in reference^60^ are shown in Table 23. It can be seen from Table 23 that the ranking results of our method are the same as those of the compared models. This shows that our method is equally effective and feasible in this case.

This subsection compares three MAGDM models for hesitant fuzzy linguistic term sets based on likelihood distributions. In two-way decision-making, we do not consider the loss incurred by experts acting on different alternatives. Instead, we use the relative closeness calculated based on the TOPSIS method to transform the model into a two-way decision model. In these cases, our method has the same results of choosing the optimal solution as these probability distribution-based models, which illustrates the excellent adaptability of our method in the MAGDM setting of hesitant fuzzy linguistic term sets, and the method can additionally take into account the loss in the decision-making process. However, our method has poor flexibility compared to these models.

### Comparative analysis with the method for group decision-making in the DSET framework

Since HFLTSs enables decision makers to use linguistic terms to express hesitancy in evaluation^11^, provides a three-way decision approach for a MAGDM problem under the HFL environment with the help of behavioral multigranularity DTRSs. In order to verify the effectiveness of the calculation method of the conditional probability of our model in the three-way decision-making model under the HFL environment, we conducted a comparative analysis with this model. In reference^11^, a different case of green suppliers is used to illustrate the method, therefore, our contrast will be in this case.

In this green supplier selection problem, four green suppliers $U = \{u_1, u_2, u_3, u_4\}$ are to be assessed relating to five criteria $P = \{p_1, p_2, p_3, p_4, p_5\}$. Three decision-makers $e_1$, $e_2$ and $e_3$ are invited to evaluate green suppliers, the evaluation results are demonstrated in Tables 5, 6 and 7 in reference^11^, based on a LTS $S = \{s_\text {-4} = extremely\ poor,\ s_{-3} = very \ poor,\ s_\text {-2} = poor,\ s_\text {-1} = slight \ poor,\ s_0 = fair,\ s_1 = slight \ good,\ s_2 = very\ good,\ s_3 = good,\ s_4 = extremely\ good\}$.

Regarding the difference in the way of obtaining the thresholds, in reference^11^ the thresholds are obtained by the calculation of the relative loss function, while in our method the thresholds are derived from the calculation of the loss function evaluated by experts. Therefore, to make a reasonable comparison, we use the thresholds in reference^11^ to compare and analyze the calculation method of conditional probability and the classification effect of the alternatives.

In reference^11^, each supplier was evaluated by three experts and obtained three pairs of thresholds and three conditional probability values. Therefore, the conditional probabilities calculated by our method are compared with the three conditional probabilities, and the results are shown in Table 24. Afterwards, the three thresholds of each supplier and the mean of the thresholds use decision rules to classify suppliers respectively. The decision results are shown in Table 25.

**Table 24:** The comparison of conditional probability.

	$e_1$	$e_2$	$e_3$	our method
$Pr( X | u_1)$	0.705	0.637	0.702	0.640
$Pr(X | u_2)$	0.622	0.588	0.641	0.344
$Pr(X | u_3)$	0.689	0.617	0.585	0.456
$Pr( X | u_4)$	0.655	0.640	0.667	0.551

**Table 25:** The comparison of decision results.

Methods	*POS*(*X*)	*BND*(*X*)	*NEG*(*X*)
Mean thresholds	$\{u_1\}$	$\{u_2,u_3,u_3\}$	$\emptyset$
Thresholds obtained by $e_1$	$\{u_1\}$	$\{u_2,u_3,u_3\}$	$\emptyset$
Thresholds obtained by $e_2$	$\{u_1\}$	$\{u_2,u_3,u_3\}$	$\emptyset$
Thresholds obtained by $e_3$	$\{u_1\}$	$\{u_3,u_3\}$	$\{ u_2 \}$
Method in reference^11^	$\{u_1\}$	$\{u_2,u_3,u_3\}$	$\emptyset$

From the decision result table, we can see that the decision results of our method under different threshold conditions are almost consistent with those in reference^11^. However, in the thresholds calculated from the evaluation information of expert $e_3$, supplier $u_2$ is classified into *NEG*(*X*) from *BND*(*X*), which is caused by the difference of conditional probability. First, the conditional probability values calculated by our method are relatively low, which is due to the fact that we combine the evaluations of multiple experts through evidence theory and take into account the information among experts, while the method in reference^11^ only considers the information of a single decision maker. Second, the TOPSIS method consider all alternatives in the same criteria, thus in our method, the calculation of alternative conditional probabilities can take advantage of more information.

According to the comparative analysis of the above examples, the advantages of our proposed 3WD model can be summed up as: First, our proposed model provides a new method for computing conditional probabilities based on DSET and TOPSIS. In this view, our proposed model takes into account the gap between the alternatives and the ideal optimal and worst alternatives under the HFL environment, enriching the theory of 3WD. Second, our proposed method shows good results in different numerical cases, and can better integrate expert opinions through DSET. In this view, our proposed method can expand the application breadth of 3WD in MAGDM problems.

## Conclusions

Considering the individual preferences and interactions of different decision makers, this paper introduces a fusion method based on Dempster-Shafer evidence theory and the TOPSIS method into the multi-attribute three-way group decision making model to solve the problem of information loss in the fusion of multi-attribute group decision making information in hesitant fuzzy linguistic environments, and to achieve better differentiation of alternatives. First, this paper uses evidence theory to establish an evidence-based decision-making information table in a hesitant fuzzy linguistic environment, and uses the TOPSIS method to calculate the conditional probability of the plan. Then, the cost function of each option is considered to calculate the threshold and derive the decision rule. Through comparative analysis of multiple examples, the method proposed in this article has good results in different numerical examples, and can better integrate expert opinions through evidence theory and improve the discrimination of alternatives. From this perspective, the method proposed in this article can expand the application scope of the three-way decision theory in uncertainty assessment information of HFL, such as intelligent decision-making of plans, classification of entities under complex information, etc. In addition, a more fine-grained representation of the evaluation linguistic terms can enable decision-makers to express more information about the evaluation object, and an information table that is more in line with the decision-maker’s preferences will be more beneficial to decision-making. On the other hand, the weight values of attributes in the three-way decision-making model proposed in this article are limited by the definition of experts. However, each decision-maker may assign attribute weights differently, so in subsequent research, decision-makers will consider the impact of different attribute preferences on decision-making, and consider improving the weight determination method to further improve the model.

## Data Availability

All data generated or analysed during this study are included in this published article .
